# Bis(dimethyl­ammonium) tetra­chlorido­dimethyl­stannate(IV)

**DOI:** 10.1107/S1600536811013584

**Published:** 2011-05-07

**Authors:** Tidiane Diop, Libasse Diop, Francois Michaud

**Affiliations:** a Laboratoire de Chimie, Universite Chekh Anta Diop, Dakar, Senegal; b Service commun d’analyse par diffraction des rayons X, Universite de Bretagne Occidentale, 6 avenue Victor Le Gorgeu, CS 93837, F-29238 BREST Cedex 3, France

## Abstract

Regular crystals of the title compound, (C_2_H_8_N)_2_[Sn(CH_3_)_2_Cl_4_], were obtained by reacting SnMe_2_Cl_2_ with (CH_3_)_2_NH·HCl in ethanol in a 1:1 ratio. The Sn atom lies on a center of symmetry and is six-coordinated. It has a distorted octahedral SnC_2_Cl_4_ environment with the Cl atoms in *cis* positions. The Cl atoms are connected to dimethyl­ammonium cations through N—H⋯Cl hydrogen bonds, forming an infinite chain extending parallel to [010].

## Related literature

For background to organotin(IV) chemistry, see: Gielen *et al.* (1996 ▶); Evans & Karpel (1985 ▶); Crowe *et al.* (1994 ▶); Diasse-Sarr *et al.* (1997) ▶; Diop *et al.* (2002, ▶ 2003) ▶. For related compounds, see: Valle *et al.* (1985 ▶); Casas *et al.* (1996 ▶); Diop *et al.* (2011 ▶).
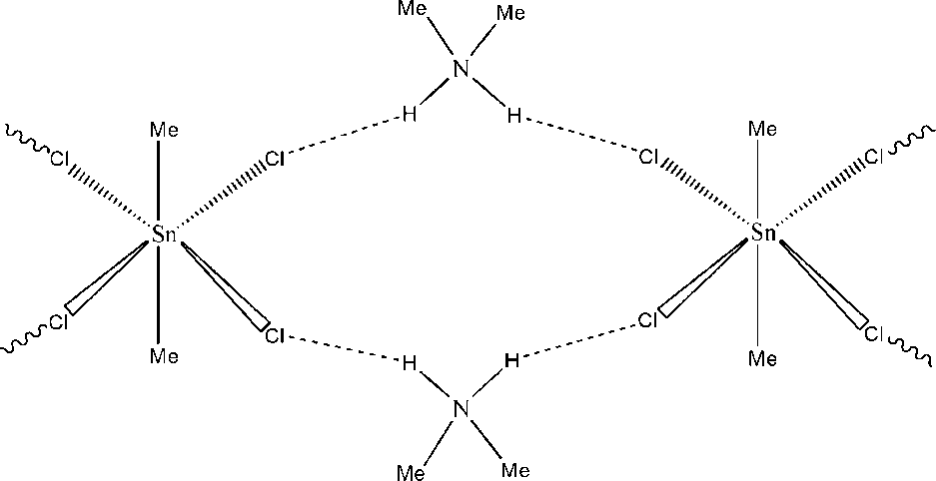


## Experimental

### 

#### Crystal data

(C_2_H_8_N)_2_[SnCH_3_)_2_Cl_4_]
*M* *_r_* = 382.75
Triclinic, 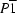

*a* = 6.6162 (9) Å
*b* = 7.3703 (11) Å
*c* = 8.4555 (12) Å
α = 109.625 (14)°
β = 98.345 (12)°
γ = 92.812 (12)°
*V* = 382.13 (9) Å^3^
*Z* = 1
Mo *K*α radiation
μ = 2.34 mm^−1^
*T* = 297 K
0.5 × 0.3 × 0.2 mm

#### Data collection

Oxford Diffraction Xcalibur Sapphire2 diffractometer
Absorption correction: multi-scan (*CrysAlis CCD*; Oxford Diffraction, 2009 ▶) *T*_min_ = 0.352, *T*_max_ = 0.652
3329 measured reflections
1871 independent reflections
1839 reflections with *I* > 2σ(*I*)
*R*_int_ = 0.018

#### Refinement

*R*[*F* ^2^ > 2σ(*F* ^2^)] = 0.023
*wR*(*F* ^2^) = 0.060
*S* = 1.06
1871 reflections
64 parameters
H-atom parameters constrained
Δρ_max_ = 0.56 e Å^−3^
Δρ_min_ = −0.43 e Å^−3^

### 

Data collection: *CrysAlis CCD* (Oxford Diffraction, 2009 ▶); cell refinement: *CrysAlis RED* (Oxford Diffraction, 2009 ▶); data reduction: *CrysAlis RED*; program(s) used to solve structure: *SIR92* (Altomare *et al.*, 1999) ▶; program(s) used to refine structure: *SHELXL97* (Sheldrick, 2008 ▶); molecular graphics: *ORTEP-3 for Windows* (Farrugia, 1997 ▶); software used to prepare material for publication: *PLATON* (Spek, 2009 ▶).

## Supplementary Material

Crystal structure: contains datablocks I, global. DOI: 10.1107/S1600536811013584/br2163sup1.cif

Structure factors: contains datablocks I. DOI: 10.1107/S1600536811013584/br2163Isup2.hkl

Additional supplementary materials:  crystallographic information; 3D view; checkCIF report

## Figures and Tables

**Table 1:** Hydrogen-bond geometry (Å, °)

*D*—H⋯*A*	*D*—H	H⋯*A*	*D*⋯*A*	*D*—H⋯*A*
N1—H1*E*⋯Cl1	0.9	2.31	3.201 (2)	169
N1—H1*D*⋯Cl2^i^	0.9	2.37	3.229 (2)	160

Symmetry code: (i) 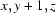.

**Fig. 1.**
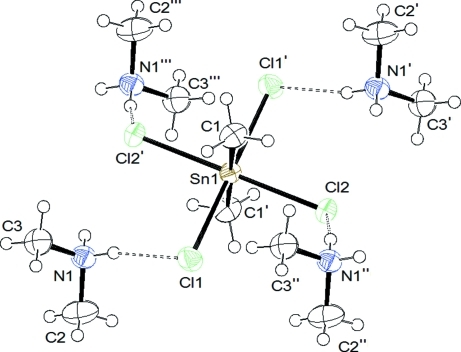
Molecular packing around one anion implying hydrogen bonds (dashed lines) with the atom numbering used and 50% probability displacement elipsoids. Symmetry operations : ['] -x, -y, -z; [''] x, y+1,z, ['''] -x, -y, -z.

## supplementary crystallographic information

### Comment

As some compounds belonging to organotin family have been screened and found to be very more active than *cis* platin towards some kinds of cancer, many groups have been involved in the seek of new organotin compounds (Gielen, 1996; Crowe, 1994). In another hand the various applications of compounds of this family have been outlined (Evans & Karpel, 1985). In our group we have yet published some papers in this field (Diop *et al.* 2002; Diop *et al.* 2003; Diasse-Sarr *et al.* 1997). In this paper we have initiated the study of the interactions between (CH_3_)_3_NH.Cl and SnMe_2_Cl_2_ which has yielded [(CH_3_)_2_NH_2_^+^]_2_[SnMe_2_Cl_4_^2-^], X-ray structure determination of which has been carried out.

In the [SnMe_2_Cl_4_]^2-^ anion the tin atom, which lies on a center of symmetry, is coordinated to the two methyl groups and four Cl atoms (Fig 1) in an octahedral geometry with *trans* methyl groups.

The Sn—C bond distances (2.116Å) are practically equal to those found in other octahedral dimethyltin(IV) diaquo-dichloro complexes SnMe_2_(H_2_O)_2_Cl_2_ (2.112 Å) reported by Valle *et al.* (1985) and longer than those in [Hthiamine][SnMe_2_(H_2_O)_2_Cl_2_]Cl (2.092 Å and 2.084 Å) reported by Casas *et al.* (1996).

The Cl—Sn—Cl and Cl—Sn—CH3 angles being very near to 90° indicates an almost perfect octahedron. The interactions between [(CH_3_)NH_2_^+^] and anion are hydrogen bonds type. The C—N—C angles of the cation is close to 109°, in agreement with the expected sp^3^ hybridation. The interactions between [(CH_3_)NH_2_^+^] and anion imply hydrogen bonds.

### Experimental

The title compound has been obtained as white crystalline solid by reacting dimethylammonium chloride (Merck) with dimethyltin dichloride (Aldrich) in ethanol (1/1 ratio, mp: 190°). After a slow solvent evaporation colourless crystals suitable for X-ray work were obtained. All the chemicals were used without any further purification.

### Crystal data

**Table:**

(C_2_H_8_N)_2_[SnCH_3_)_2_Cl_4_]	*Z* = 1
*M**_r_* = 382.75	*F*(000) = 190
Triclinic, *P*1	*D*_x_ = 1.663 Mg m^−^^3^
Hall symbol: -P 1	Mo *K*α radiation, λ = 0.71073 Å
*a* = 6.6162 (9) Å	Cell parameters from 3675 reflections
*b* = 7.3703 (11) Å	θ = 2.9–31.3°
*c* = 8.4555 (12) Å	µ = 2.34 mm^−^^1^
α = 109.625 (14)°	*T* = 297 K
β = 98.345 (12)°	Fragment of rounded block, colourless
γ = 92.812 (12)°	0.5 × 0.3 × 0.2 mm
*V* = 382.13 (9) Å^3^	

### Data collection

**Table:**

Oxford Diffraction Xcalibur Sapphire2 diffractometer	1871 independent reflections
Radiation source: sealed X-ray tube	1839 reflections with *I* > 2σ(*I*)
graphite	*R*_int_ = 0.018
Detector resolution: 8.3622 pixels mm^-1^	θ_max_ = 28.3°, θ_min_ = 4.1°
4 stepped ω–scans over 115 deg. with kappa –79 deg. (chi –58.3 deg.), phi 0, 90, 180, 270 deg. step 1 deg., exposure time 45 s detector distance 50 mm detector angle 30 deg.	*h* = −8→7
Absorption correction: multi-scan (*CrysAlis CCD*; Oxford Diffraction, 2009)	*k* = −9→9
*T*_min_ = 0.352, *T*_max_ = 0.652	*l* = −11→11
3329 measured reflections	

### Refinement

**Table:**

Refinement on *F*^2^	Primary atom site location: structure-invariant direct methods
Least-squares matrix: full	Secondary atom site location: difference Fourier map
*R*[*F*^2^ > 2σ(*F*^2^)] = 0.023	Hydrogen site location: inferred from neighbouring sites
*wR*(*F*^2^) = 0.060	H-atom parameters constrained
*S* = 1.06	*w* = 1/[σ^2^(*F*_o_^2^) + (0.0362*P*)^2^ + 0.2233*P*] where *P* = (*F*_o_^2^ + 2*F*_c_^2^)/3
1871 reflections	(Δ/σ)_max_ < 0.001
64 parameters	Δρ_max_ = 0.56 e Å^−^^3^
0 restraints	Δρ_min_ = −0.43 e Å^−^^3^

### Special details

**Table:**

Geometry. All e.s.d.'s (except the e.s.d. in the dihedral angle between two l.s. planes) are estimated using the full covariance matrix. The cell e.s.d.'s are taken into account individually in the estimation of e.s.d.'s in distances, angles and torsion angles; correlations between e.s.d.'s in cell parameters are only used when they are defined by crystal symmetry. An approximate (isotropic) treatment of cell e.s.d.'s is used for estimating e.s.d.'s involving l.s. planes.
Refinement. Refinement of *F*^2^ against ALL reflections. The weighted *R*-factor *wR* and goodness of fit *S* are based on *F*^2^, conventional *R*-factors *R* are based on *F*, with *F* set to zero for negative *F*^2^. The threshold expression of *F*^2^ > σ(*F*^2^) is used only for calculating *R*-factors(gt) *etc*. and is not relevant to the choice of reflections for refinement. *R*-factors based on *F*^2^ are statistically about twice as large as those based on *F*, and *R*- factors based on ALL data will be even larger.

### Fractional atomic coordinates and isotropic or equivalent isotropic displacement parameters (Å^2^)

**Table:**

	*x*	*y*	*z*	*U*_iso_*/*U*_eq_	
C1	0.2689 (4)	0.3818 (4)	0.5967 (3)	0.0404 (5)	
H1A	0.2902	0.2502	0.5847	0.061*	
H1B	0.1366	0.3849	0.5343	0.061*	
H1C	0.275	0.4566	0.715	0.061*	
Cl1	0.25609 (10)	0.40746 (9)	0.20155 (7)	0.04289 (14)	
Cl2	0.64584 (8)	0.16207 (8)	0.39707 (7)	0.03603 (12)	
Sn1	0.5	0.5	0.5	0.02900 (8)	
C2	0.2993 (5)	0.8184 (6)	0.0309 (4)	0.0621 (8)	
H2A	0.4272	0.764	0.0166	0.093*	
H2B	0.3073	0.9423	0.0167	0.093*	
H2C	0.1905	0.7333	−0.0527	0.093*	
C3	0.0570 (4)	0.9131 (4)	0.2302 (4)	0.0479 (6)	
H3A	0.0331	0.9174	0.3406	0.072*	
H3B	−0.0493	0.8274	0.1442	0.072*	
H3C	0.0563	1.0407	0.2239	0.072*	
N1	0.2581 (3)	0.8422 (3)	0.2023 (3)	0.0381 (4)	
H1D	0.3576	0.9263	0.28	0.046*	
H1E	0.2622	0.7276	0.2183	0.046*	

### Atomic displacement parameters (Å^2^)

**Table:**

	*U*^11^	*U*^22^	*U*^33^	*U*^12^	*U*^13^	*U*^23^
C1	0.0393 (11)	0.0380 (11)	0.0450 (12)	−0.0035 (9)	0.0167 (10)	0.0128 (10)
Cl1	0.0486 (3)	0.0369 (3)	0.0360 (3)	−0.0001 (2)	−0.0059 (2)	0.0094 (2)
Cl2	0.0387 (3)	0.0282 (2)	0.0402 (3)	0.00715 (19)	0.0087 (2)	0.0093 (2)
Sn1	0.03107 (11)	0.02496 (11)	0.03074 (11)	−0.00012 (7)	0.00626 (7)	0.00940 (8)
C2	0.0604 (18)	0.078 (2)	0.0417 (14)	−0.0007 (16)	0.0195 (13)	0.0098 (14)
C3	0.0387 (12)	0.0550 (15)	0.0478 (14)	0.0036 (11)	0.0083 (10)	0.0151 (12)
N1	0.0382 (10)	0.0390 (10)	0.0351 (9)	0.0007 (8)	0.0029 (7)	0.0120 (8)

### Geometric parameters (Å, °)

**Table:**

C1—Sn1	2.116 (2)	C2—H2A	0.96
C1—H1A	0.96	C2—H2B	0.96
C1—H1B	0.96	C2—H2C	0.96
C1—H1C	0.96	C3—N1	1.475 (3)
Cl1—Sn1	2.6441 (7)	C3—H3A	0.96
Cl2—Sn1	2.6297 (7)	C3—H3B	0.96
Sn1—C1^i^	2.116 (2)	C3—H3C	0.96
Sn1—Cl2^i^	2.6297 (7)	N1—H1D	0.9
Sn1—Cl1^i^	2.6441 (7)	N1—H1E	0.9
C2—N1	1.468 (4)		
			
Sn1—C1—H1A	109.5	Cl1^i^—Sn1—Cl1	180
Sn1—C1—H1B	109.5	N1—C2—H2A	109.5
H1A—C1—H1B	109.5	N1—C2—H2B	109.5
Sn1—C1—H1C	109.5	H2A—C2—H2B	109.5
H1A—C1—H1C	109.5	N1—C2—H2C	109.5
H1B—C1—H1C	109.5	H2A—C2—H2C	109.5
C1^i^—Sn1—C1	180.00 (13)	H2B—C2—H2C	109.5
C1^i^—Sn1—Cl2^i^	90.42 (7)	N1—C3—H3A	109.5
C1—Sn1—Cl2^i^	89.58 (7)	N1—C3—H3B	109.5
C1^i^—Sn1—Cl2	89.58 (7)	H3A—C3—H3B	109.5
C1—Sn1—Cl2	90.42 (7)	N1—C3—H3C	109.5
Cl2^i^—Sn1—Cl2	180	H3A—C3—H3C	109.5
C1^i^—Sn1—Cl1^i^	90.43 (8)	H3B—C3—H3C	109.5
C1—Sn1—Cl1^i^	89.57 (8)	C2—N1—C3	112.7 (2)
Cl2^i^—Sn1—Cl1^i^	89.90 (2)	C2—N1—H1D	109.1
Cl2—Sn1—Cl1^i^	90.10 (2)	C3—N1—H1D	109.1
C1^i^—Sn1—Cl1	89.57 (8)	C2—N1—H1E	109.1
C1—Sn1—Cl1	90.43 (8)	C3—N1—H1E	109.1
Cl2^i^—Sn1—Cl1	90.10 (2)	H1D—N1—H1E	107.8
Cl2—Sn1—Cl1	89.90 (2)		

Symmetry codes: (i) −*x*+1, −*y*+1, −*z*+1.

### Hydrogen-bond geometry (Å, °)

**Table:**

*D*—H···*A*	*D*—H	H···*A*	*D*···*A*	*D*—H···*A*
N1—H1E···Cl1	0.9	2.31	3.201 (2)	169
N1—H1D···Cl2^ii^	0.9	2.37	3.229 (2)	160

Symmetry codes: (ii) *x*, *y*+1, *z*.
